# Malacosteon, Mollities Ossium, Osteo Malacia

**Published:** 1898-03

**Authors:** R. E. Graul

**Affiliations:** Brenham, Texas


### TEXAS MEDICAL JOURNAL.

ESTABLISHED JULY, 1885.

PUBLISHED MONTHLY.—SUBSCRIPTION $2.00 A YEAR.

VOL. XIII. AUSTIN, MARCH, 1898. No. 9.

### Original Contributions.

For the Texas Medical Journal.

### Malacosteon, Mollities Ossium, Osteo Malacia.

R. E. GRAUL, M. D., BRENHAM, TEXAS.

[Read before the Brenham Medical Society, June 1, 1897.]

The subject which I intend to discuss before you is a disease in which the bones of the skeleton become by degrees decalcified, so that they no longer can support the weight of the body, but either bend or break. Strange to say, like malignant acute anæmia, this disease attacks almost exclusively women during the child-bearing period; and it is apparent to every close observer, that there is an intimate connection between the gravid state and osteo malacia—a veritable predisposition to it. This predisposition is distinctly increased with every new pregnancy, which is borne out by the fact that multiparæ, generally at the age between 30 to 40, are mostly the victims. Some did, until lately, regard and classify this disease as an atrophy; modern investigations have, however, proven the fact that it is not an atrophy, but a decalcification; a gradual disappearance of all calcareous matter from the bony structure, rendering it soft and brittle. Others have confounded it with rheumatism, especially in its earlier stages, and ascribed its origin to an excess of lactic acid in the blood. This rheumatic theory is, however, exploded by the fact: that the pains, even in the beginning of the trouble, are distinct osteocopic and not rheumatic, which means to say:

the pain is somewhat analogous to the pain of real ostitis in character as well as in severity. It is, perhaps, this osteocopic character of the pain connected with the disease, which has led some to fall into another error and define malacosteon as an inflammatory process going on in the bone substance. To fully understand the gravity of such cases, we must look into the anatomical character of this formidable affection.

I have said that in mollities ossium the bone becomes decalcified by degrees. This change is spreading from within outwards, until a mere shell of compact tissue is left. This shell is never absorbed—all autopsies have evidenced this. The medullary cavity enlarges until the interior becomes a cheesy gelatenous mass enclosed in a periosteal shell, no longer capable of rendering any support to the body. How can we diagnose such a case?—a correct diagnosis being the only thing of comfort to a physician in such otherwise absolutely hopeless cases; nay, not only a comfortable thing, but an item of necessity—a means of self-preservation of the attending doctor's prestige. If we diagnose a case so serious as rheumatism,—as this is generally done, and hold out a promise of cure, and later on some one else makes a correct statement of the case, we are, to say the least, immortally blamed; besides we are more than apt to lose the confidence of the patient and his family. Let us, then, in as much as we are not able to cure, direct all our efforts to diagnose correctly. How are we, in such a given case, able to make a correct diagnosis? There is but one way, and that is: a diagnosis of such a case can and should be made by *exclusion* only. Exclude those maladies which closely resemble mollities ossium; exclude them by a minute and careful comparison, and the chances are all in favor of you. Neglect this precaution, and in nine cases out of ten you will most beautifully fool yourself. Don't jump at rheumatism —the one thing with which the general practitioner is apt to confound a disease like this. Remember, you have plenty of time to observe your patient; perhaps many days—utilize this time. If she is a multiparæ—apparently well preserved—if she has no rheumatic antecedents, and I might state right here, no syphillitic antecedents; if the pains are principally of a nocturnal character—always aggravated at night—confined to special regions, the spine, hip, legs, then be extremely solicitous in telling her specifically the nature of her ailment. Wait! The policy of waiting is true wisdom in many things, especially here. If it is rheumatism this pain will disappear—especially under proper treatment—but suppose it does not yield to such a treatment? The pains of malacosteon never cease and never yield to anything. Another point: rheumatic pains are, when acute and intense, accompanied by high fever. Here in malacosteon is absence of fever. Then again, the pains of mollitis are always in the same place; they are deep-seated; they are in the bones, and by calling the patient's attention to this characteristic she will tell you so; they are osteocopic pains; their very character is such that upon prolonged and careful observation you cannot well err. Yes, but could not this osteocopic character again mislead us and diagnose an ostitis? That is true, but when we remember that an ostitis has a very acute beginning; that it rarely attacks many bones in different places of the skeleton, but as a rule, the long bones of one or the other extremity; that the pain is removed by strong pressure. If we remember these points of interest we surely will avoid this snag. Now then, by circumventing rheumatism and excluding ostitis, we have arrived at a point where our path to a correct diagnosis will be free from obstructions.

There is another fortunate element for the poor doctor in such cases, that is the circumstance that in most cases of such nature the city doctor is only called in after a half dozen or more other fellows have seen the case and given vent to their blessed ignorance. If that is the case, remember the long duration of the patient's illness—another pointer; malacosteon will extend over a period of from 2 to 4 years!

If the case is in anyway advanced you will be able to feel the enormous size of the bones, the patient of course not being able to walk or even sustain the weight of her body in bed in the sitting posture. Here the probabilities are that upon any movement a fracture will be established, and if the patient permit, you may at will, by merely pressing upon the shaft of a long bone, produce a fracture without pain. If that can be done, then of course the diagnosis is evident. In well advanced cases the entire body becomes shapeless, dislocations of vertebræ, fracture of ribs, of the scapulæ, of the long bones, are nothing uncommon; the patient is helpless and bedridden until death from exhaustion relieves her and her attendants. Strange that during all the prolonged struggle a constitutional cachexia—such as in other severe chronic diseases—is never developed. The prognosis is exceedingly unfavorable. No remedial agents yet discovered have ever arrested the course of this dreadful disease or effected a cure. We are simply helpless in the face of the enemy.

I remember two cases which have come under my observation, which I herewith shall quote to you from my note book, as an illustration upon the subject:

Case 1 was a lady in St. Louis, which I was permitted to see through the courtesy of medical friends, aged 42 years, and a mother of five children. Mollities ossium had developed after her last child-birth, which had been an instrumental delivery. She had been ill one year and seven months when I first saw her. All of her bones, as far as discernable, were affected, countless fractures were sustained; so soft were her bones that even the most careful lifting with the sheet on which she lay by trained attendants would invariably result in one or more factures. Four more months she lingered in this dreadful condition until relief came.

The other case was observed here in our county during January of last year (1896). She, too, was multiparus, had a previous history of syphilis, which probably played an important role in this particular case. In her case the femurs of both legs being principally attacked, softened to such a degree that they could be bent in any direction. She, too, made an *esitus lethalis* after much suffering, and in conclusion I might say that both cases were in the beginning promptly diagnosed as cases of rheumatism.

